# RNA Interference of Myocyte Enhancer Factor 2A Accelerates Atherosclerosis in Apolipoprotein E-Deficient Mice

**DOI:** 10.1371/journal.pone.0121823

**Published:** 2015-03-20

**Authors:** Wen-ping Zhou, Hui Zhang, Yu-xia Zhao, Gang-qiong Liu, Jin-ying Zhang

**Affiliations:** 1 Department of Cardiology, the First Affiliated Hospital of Zhengzhou University, Zhengzhou, Henan, P.R. China; 2 Department of Medical Equipment, the First Affiliated Hospital of Zhengzhou University, Zhengzhou, Henan, P.R. China; The University of Tennessee Health Science Center, UNITED STATES

## Abstract

**Objective:**

Myocyte enhancer factor-2A (MEF 2A) has been shown to be involved in atherosclerotic lesion development, but its role in preexisting lesions is still unclear. In the present study we aim to assess the role of MEF 2A in the progression of pre-existing atherosclerosis.

**Methods:**

Eighty apolipoprotein E-deficient mice (APOE KO) were randomly allocated to control, scramble and MEF 2A RNA interference (RNAi) groups, and constrictive collars were used to induce plaque formation. Six weeks after surgery, lentiviral shRNA construct was used to silence the expression of MEF 2A. Carotid plaques were harvested for analysis 4 weeks after viral vector transduction. Inflammatory gene expression in the plasma and carotid plaques was determined by using ELISAs and real-time RT-PCR.

**Results:**

The expression level of MEF 2A was significantly reduced in plasma and plaque in the RNAi group, compared to the control and NC groups, whereas the expression level of pro-inflammatory cytokines was markedly increased. Silencing MEF 2A using lentiviral shRNA significantly reduced the plaque collagen content and fibrous cap thickness, as well as increased plaque area. However, silencing MEF 2A had no obvious effect on plaque lipid content.

**Conclusions:**

Lentivirus-mediated MEF 2A shRNA accelerates inflammation and atherosclerosis in APOE KO mice, but has no effect on lipoprotein levels in plasma.

## Introduction

Atherosclerosis and its clinical complications are the leading causes of death and disability in the western world. It has been increasingly recognized that genetic factors play an important role in the development of atherosclerosis. The myocyte enhancer factor 2A (MEF 2A) gene is known to play diverse roles in atherosclerotic plaque formation and thrombosis, as well as in the morphogenesis and myogenesis of cardiac, skeletal and smooth muscle cells by controlling cell proliferation and apoptosis[1,2]. In 2003, A mutant MEF 2A with a 21 base pair (bp) deletion in the exon 11 was found to cause coronary heart disease (CHD) with autosomal- dominant pattern of inheritance [3]. Latter, three genetic variants of MEF 2A including N263S, P279L, and G283D were found in sporadic patients with CHD, suggesting that MEF 2A plays a pivotal role in the pathogenesis of atherosclerosis in non-familial cases [4]. These studies implicate that those genetic variants are causative for atherosclerosis/CHD [3]. However, subsequent investigations failed to confirm MEF 2A as a causal gene of CHD or detect the presence of this mutation [9], which contradicts previous findings [5–8]. Several lines of evidences demonstrate that there is no evidence of an association between MEF 2A genetic variants and the risk of atherosclerosis. In addition, the abovementioned mutations in MEF 2A were observed in clinically unaffected control subjects and did not segregate with atherosclerosis. So the exact role of MEF 2A in the progression of atherosclerosis remains unclear.

RNA interference (RNAi) has been shown to be quite efcacious in silencing target genes in animal models of atherosclerosis. In the present study, we investigated the progression of atherosclerosis by examining inflammation in apolipoprotein E-deficient mice (APOE KO) following delivering MEF2A shRNA and found that silencing MEF 2A accelerates atherosclerosis.

## Methods

### Cell culture

Mice aortic endothelial cells (MAECs) were purchased from ATCC and routinely cultured in DMEM containing 10% FBS, 100ug/ml Ampicillin and 100U/ml Streptomycin.

### Lentiviral vector production

To silence MEF2A expression, lentiviral shRNA vectors were constructed using 4 different shRNA sequences against MEF 2A including 5'-GCAGTTATCTCAGGGTTCAAA-3'(A), 5'- CCAAATACTGAGGACAGAGAA-3'(B), 5'- CCAGCTCAACATTAGCAGATT-3' (C)and 5'- CCCTTAATGAATTGATGACTA-3'(D). A scrambled control shRNA lentiviral vector was also constructed using target sequences 5'-TTCTCCGAACGTGTCACGT-3' (Jerui-Bioscience, Shanghai, CHINA). Lentiviral vectors were produced in HEK293 cells as previously described [10–12]; Virus titers was 1 × 10^9^ TU (transduction units)/mL as determined by examining green fiuorescent protein (GFP). Four lentiviral shRNAs against MEF 2A and scramble shRNA vector were used to transduce the MAECs at a multiplicity of infection (MOI) of 50. To screen the target for the most effective gene knockdown, transduced MAECs were collected for western blot and real-time PCR at day 4 following transduction.

### Animals and experimental protocol

Eighty male APOE KO mice were obtained from the Beijing University Animal Research Center. All animal work has been approved by the Institutional Committee of Animal Care and Use of Zhengzshou University. Mice were bred and maintained in the animal center of Zhengzhou University. Eighty male APOE KO mice received a high-fat diet (0. 25% cholesterol and 15% cocoa butter). Animals underwent constrictive collar placement around the left common carotid artery after anaesthesia with an intraperitoneal injection of pentobarbital sodium (30–50 mg/kg), using the techinique of von der Thüsen et al [13]. In brief, the common carotid arteries were dissected and a constrictive silastic collar (0. 30 mm) was placed on the left common carotid artery by placement of 3 circumferential silk ties [14].

Mice were randomly divided into the control (*n* = 24), scramble (*n* = 32) and MEF 2A shRNA group (*n* = 24). At the end of week 6, the carotid collars were removed and lentivirus (5×10^7^ TU, RNAi group), NC lentivirus (5×10^7^ TU, NC group) or PBS (control group) was instilled around the left common carotid artery. To investigate the transduction efficiency, two mice from the NC group were sacrificed every week after transduction (week 6). At the end of the experiment,the mice were euthanized with a lethal dose of pentobarbital sodium injected intravenously. Cryosections were viewed by fluorescence microscopy to visualize GFP expression. The remaining 72 animals were sacrificed at week 10, and the left common carotid arteries were collected for histopathological analysis.

### Histological analysis

The left common carotid artery was carefully excised and perfused by 4% formaldehyde, embedded in O. C. T. compound [14,15]. The carotid artery was cross-sectioned and stained with hematoxylin and eosin (HE),Masson’s trichrome and oil red O (ORO) staining[14,15]. Image analysis system was used for quantitative measurements (Image-Pro Plus 5. 0; MediaCybernetics, Silver Spring, MD).

### RNA extraction and real-time PCR

Total RNA was extracted from left common carotid artery homogenates with Trizol reagent. Reverse transcription (RT) was performed following the manufacturer’s instructions (CoWin-Bioscience, Beijing, CHINA). SYBR Green RT-PCR was conducted using ABI Prism 7500 Sequence Detection System (PE Applied Biosystems, Foster City, CA, USA). GAPDH was employedas an internal control. The specific primers used were as follows: 5'- TCCTCATCTGCTGTCCTGAC-3' and 5'- GAGACCACAGAGAGGAGAGC-3' for MEF 2A; 5'-GCTCAGCCAGATGCAGTTAACG-3' and 5'-TCTTGGGGTCAGCACAGACCTC-3' for monocyte chemotactic protein-1 (MCP-1); 5'-GCCTGACTCTGGTGATTTCTTG-3' and 5'-TGTTGATGTCTGCTTCTCCCTG-3' for matrix metalloproteinase-8 (MMP-8); 5'-TGTCTACTGAACTTCGGGGTGA-3' and 5'-TGGTTTGCTACGACGTGGGCTA-3' for tumor necrosis factor-α (TNF-α) and 5'-GGTGAAGGTCGGTGTGAACG-3' and 5'-CTCGCTCCTGGAAGATGGTG-3' for GADPH (Jerui-Bioscience, Shanghai, CHINA). The relative gene expression levels were calculated by using the 2^-ΔΔCt^ method [14].

### Western blot analysis

For Western blot analysis, equal amounts of protein from MAECs or tissues were separated on sodium dodecyl sulfate (SDS)–14% polyacrylamide gels and transferred to nitrocellulose membrane. After blocking with 5% nonfat milk, the blots were washed with phosphate-buffered saline (PBS) containing 0.1% Tween 20 and incubated with antibodies against MEF 2A and GAPDH at 4℃ overnight, the blots were rinsed with Tris-buffered saline plus Tween 20 (TBST) and probed with respective secondary antibody conjugated to horseradish peroxidase (diluted 1:1000) and then washed again. Subsequently, the blots were visualized by enhanced chemiluminescence. The X-ray films were scanned, and quantitative analysis was performed by ImageJ software. The housekeeping gene GAPDH was quantified as an internal control.

### Plasma lipid analysis

Plasma was acquired through centrifugation of the blood samples at 1,500 g, at 4°C and then stored at -20°C for further analysis. Expression of MEF 2A, MMP-8, TNF-α, MCP-1, and lipoprotein levels, including low-density lipoprotein cholesterol(LDL-C), high-density lipoprotein cholesterol(HDL-C), total cholesterol(TC), and triglyceride(TG)in plasma were measured using quantitative sandwich enzyme immunoassay (commercial ELISA kits) following the manufacture’s protocol (CoWin-Bioscience, Beijing, CHINA).

### Statistical analysis

Data were presented as mean ± SD. After testing the normal distribution of variables, data were compared among different groups using one-way analysis of variance (ANOVA) followed by the Student-Newman-Keuls (SNK) test for post-hoc comparisons. All statistical analyses were performed using SPSS 16. 0 software (SPSS, Chicago, IL, USA). *P* < 0.05 was considered statistically significant.

## Results

### Silencing MEF 2A expression in MAECs using lentiviral vectors

**Fig 1:**
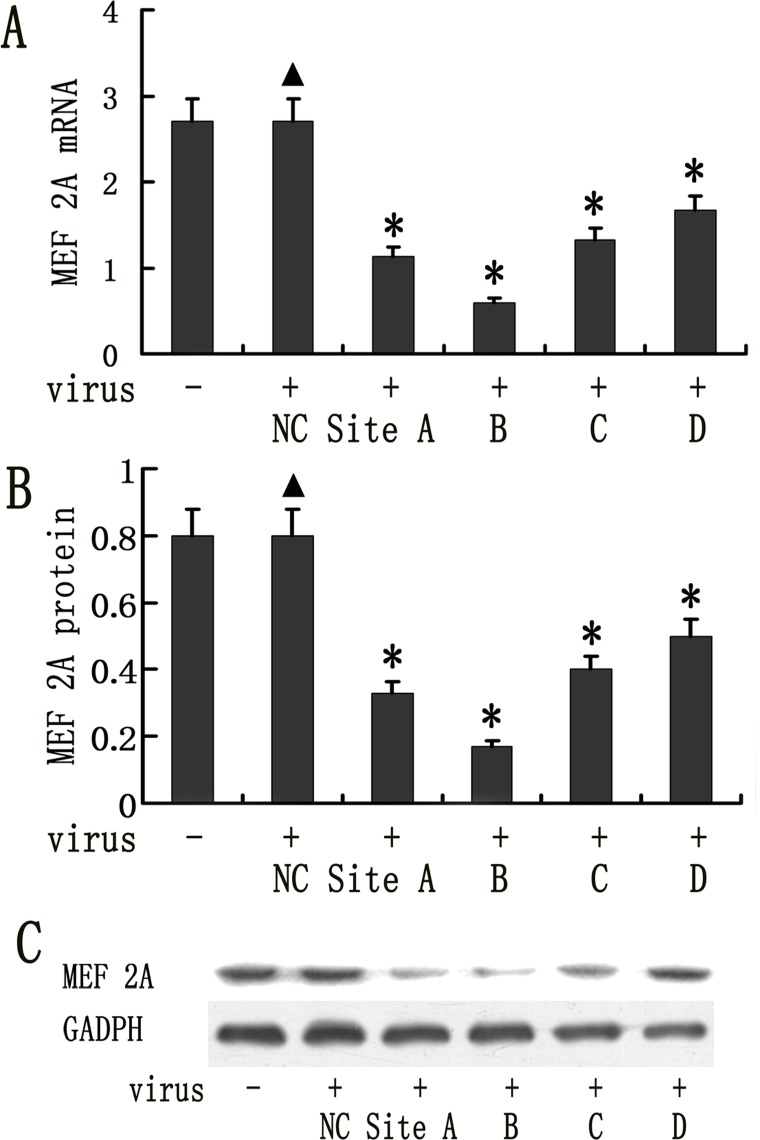
Lentiviral vector mediated knockdown of MEF 2A in mice aortic endothelial cells (MAECs). MAECs were transduced with 50 MOI of 4 different shRNA vectors and MEF 2A expression was measured on day 4 by real time RT-PCR following transduction. The GAPDH was used as an internal control. (A). MEF 2A mRNA expression detected by realtime RT-PCR (*P < 0.05) (B) MEF 2A protein expression in lentivral shRNA transduced MAECs was quantified using Image J. (C) MEF2A expression in lentiviral shRNA vector transduced MAECs was determined by Western blots. Data were presented in mean±SD. *P < 0.05. (n = 8)

The MAECs were transduced with lentiviral vectors expressing four different MEF 2A shRNAs. MEF2A expression was analyzed using real-time PCR and Western blot at 96h following transduction. MEF 2A-shRNA B was the most effective and leads to approximately 79.1% reduction in MEF 2A mRNA expression detected by real-time PCR as shown in Fig. 1A and a 66.9% reduction in MEF 2A protein expression in MAECs compared to MEF2A Scramble control (Fig. 1B-C). MEF2A shRNA A, C, and D were less efficient, which lead to 59.1, 50.6, and 38.1%, reduction at mRNA expression level, and 48.6, 42.1, 37.7% at protein level. Therefore, MEF 2A-shRNA B lentiviral vector was selected for further study.

### Silencing MEF2A expression *in vivo* using lentiviral shRNA vector

**Fig 2:**
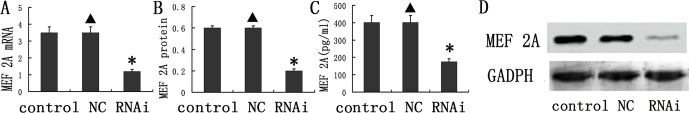
Knockdown of MEF 2A in vivo. (A) mRNA expression of MEF 2A in the plaques of the control, NC, and MEF 2A shRNA groups at week 10; (B) Protein expression of MEF 2A in the plaques of the control, NC, and shRNA groups; (C) The concentration MEF 2A in the plasma; (D) Representative Western blots used for quantification. Data are presented in the mean ± SD. * P < 0.05

We examined the mRNA and protein expression of MEF 2A in the carotid plaques, as well as the MEF 2A expression in the plasma following lentiviral vector delivery. MEF 2A mRNA and protein expression was statistically reduced in plasma of APO E KO mice in the MEF 2A knockdown group compared to scramble control and NC groups (Fig. 2). MEF 2A mRNA expression was reduced by 68.6% (*P* < 0.01, Fig. 2A), while the MEF 2A protein was decreased by 60.5% (*P* < 0.01, Fig. 2B and D) and the plasma concentration of MEF 2A was lowered by 56.4% (*P* < 0.05, Fig. 2C), compared to those in the scramble control and NC groups. In contrast, the NC group did not differ from the control group in MEF 2A expression.

**Fig 3:**
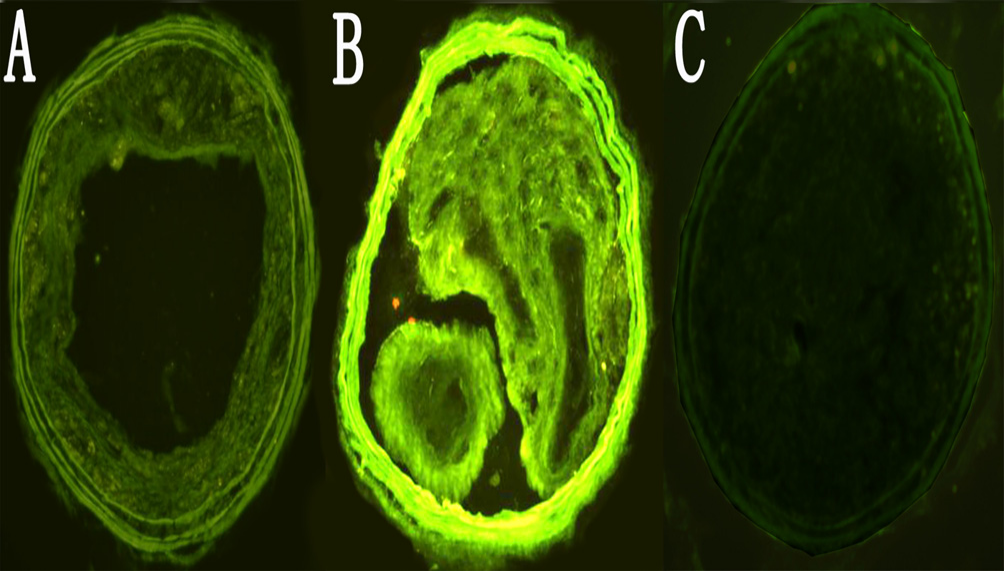
Efficiency of lentviral shRNA vector transduction in the carotid plaques. A, B, C. GFP expression in sections of the carotid plaques was imaged from NC group. Carotid plaques at 1, 2 and 4 weeks following transduction were visualized under fluorescent microscope. (200×).

Previous studies [14] have indicated that GFP expression provided an efficient and convenient approach to detect the efficiency of transduction. GFP fluorescence in carotid plaques was observed one week after transduction. The strongest GFP fluorescence was displayed two weeks after transduction (Fig. 3A-C), demonstrating that lentivirus can efficiently transduce plaques *in vivo*.

### Bodyweight and plasma lipid profiles

We observed no significant difference in the bodyweight among all groups, demonstrating that lentiviral mediated gene knockdown of MEF2A did not affect animal growth. Furthermore, TC, TG, HDL-C and LDL-C levels in plasma among all groups were not significant different, indicating that knockdown of MEF2A in plaque did not affect the plasma lipid profile (Table 1).

**Table 1:** Body weight, plasma TC, TG, HDL-C and LDL-C levels among all groups.

BW (g)	TC (mmol/L)	TG (mmol/L)	HDL-C (mmol/L)	LDL-C (mmol/L)	
Control	28.1 ± 2.9	28.2 ± 3.6	3.3 ± 0.7	3.7±0.6	31.7±4.1
NC	27.9 ± 3.5	29.3 ± 3.2	3.1 ± 0.9	3.6±0.4	29.8±3.8
RNAi	28.3 ± 3.3	28.8 ± 3.5	3.2 ± 0.8	3.9±0.5	32.4±4.5

Data are reported as the mean± SD. *P* > 0.05 among all groups. BW = body weight; TC = total cholesterol; TG = triglyceride; HDL-C = high-density lipoprotein cholesterol; LDL-C = low-density lipoprotein cholesterol; NC = negative control group. Control = control group; RNAi = RNA interference group.

### Silencing MEF2A in plaque upregulates inflammatory marker genes

**Fig 4:**
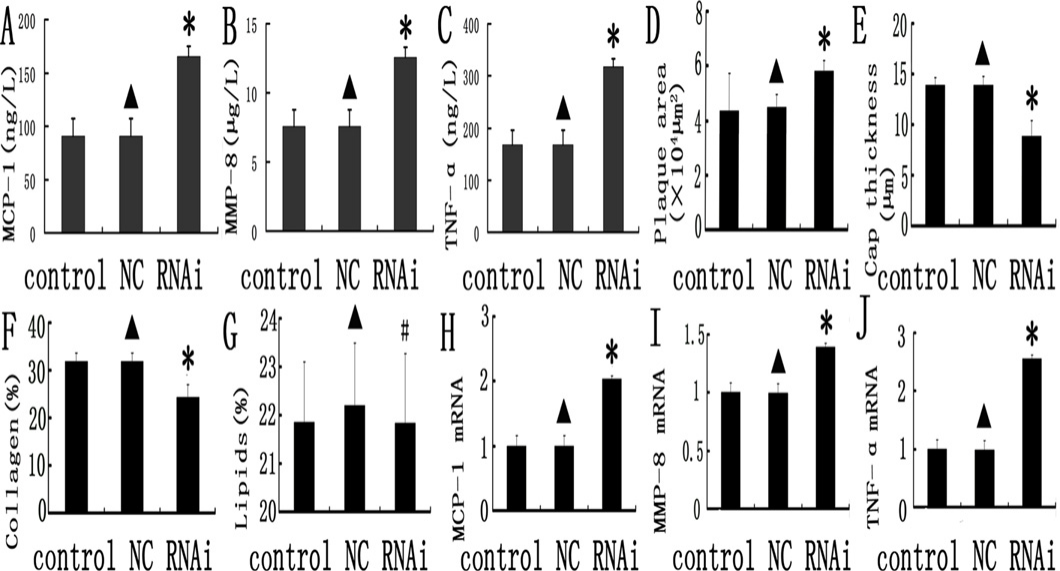
Inflammation gene expression and comparison of relative composition of plaques in the control, NC and MEF 2A RNAi groups. A, B, and C: inflammatory markers (MCP-1, MMP-8 and TNF-α) in the control, NC, and MEF 2A shRNA groups; D, E,F and G plaque morphology in the control, NC, and MEF 2A shRNA groups; Plaque area (D), Fibirious cap thickness (E),collage content (F) and lipid content (G) were shown for the control, NC and RNAi groups. H, I and J: MCP-1 (H), MMP-8 (I) and TNF-α (J) mRNA expression in carotid plaques of the control, NC and shRNA groups at week 10 was detected by real-time PCR. Data were expressed as the mean ± SD. * *P* < 0.05.

Several inflammation associated genes including MCP-1(Fig. 4A), MMP-8(Fig. 4B) and TNF-α(Fig. 4C) were detected in plasma and all of them are dramatically higher in MEF2A shRNA than the scramble control and NC groups (*P* < 0.05). These biomarkers can be used to monitor the instability of the plaque. In addition, there were no significant differences on these inflammatory markers between the scramble control and NC group (*P* > 0.05). Our data demonstrated that silencing MEF 2A in plaques promotes inflammation in mice.

### Silencing MEF2A accelerates the formation of atherosclerotic plaques

**Fig 5:**
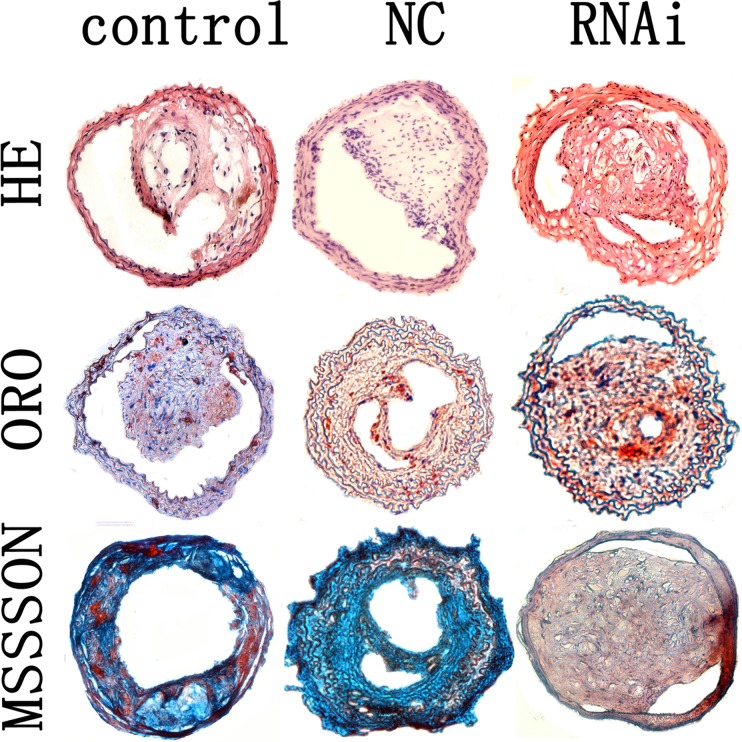
Carotid plaques in the control, NC and MEF 2A RNAi groups. Cross-sections of plaques in the control, NC and RNAi groups were stained with HE, ORO and masson’s trichrome. magnification 200×.

The plaque area in cross-section from the MEF 2A shRNA group is 58.7 ± 8.4 × 10^3^ μm^2^, which was statistical higher than the control and NC groups(42.3 ± 6.5 × 10^3^ μm^2^ and 43.7 ± 7.1 × 10^3^ μm^2^; *P* < 0.01, Fig. 4D). Fibrous cap thickness was significant lower in the MEF 2A shRNA group (8.63 ± 0.92μm) than that in the control and NC groups (12.89 ± 1.75 and 13.29 ± 1.55 μm; *P* < 0.01, Fig. 4E). As expected, no obvious differences in plaque area and fibrous cap thickness were found between the scramble control and NC groups (Figs. 4D-G and 5).

The relative content of collagen and lipids in carotid plaques were determined by histological staining. The relative content of collagen in plaques of the control, NC and RNAi groups was 30.9, 31.4, and 23.8%, and was remarkably lower in the MEF 2A shRNA group than that of the control and NC groups (*P* < 0.05, Figs. 4F and 5).

The relative content of lipids in plaques of the MEF 2A shRNA group (21.9%) was not statistically significant (*P* > 0.05) than that of the control(21.8%)and NC groups (22.3%, Fig. 4G). In addition, no significant differences in the cross-sectional plaque area, fibrous cap thickness and collagen content were found between the control and NC group (Figs. 4D-G and 5). Taken together, these data indicated that knockdown of MEF 2A in carotid plaque led to higher plaque area and lower collagen content and fibrous cap thickness than that in the control and NC groups.

## Discussion

In the present work, we assessed the effects of lentivirus-mediated MEF 2A shRNA on the progression of atherosclerotic plaque and associated inflammatory process following collar-induced atherosclerosis in APOE KO mice. The major finding of the current investigation was that knockdown of MEF2A upregulated local inflammatory cytokine expression, reduced fibrous cap thickness, decreased collagen content in plaques and increased atherosclerotic plaque areas and vulnerability. To the best of our knowledge, this is the first report to delineate the effects of MEF 2A shRNA in the progression and stabilization of atherosclerotic plaques in APOE KO mice.

It is well known that inflammation plays an important role in the progression of atherosclerosis. Major clinical complications arise when atherosclerotic plaques evolve into complex, unstable forms characterized by a thin fibrous cap, a large lipid-filled necrotic core and an accumulation of macrophages. Atherosclerosis is caused in part by genetic factors. Substantial studies demonstrated that the MEF 2A gene played a pivotal role in atherosclerotic plaque formation and plaque rupture, as well as in morphogenesis and myogenesis of cardiac, skeletal and smooth muscle cells and the control of cell growth, survival and apoptosis[1,2,16–19]. In fact, the exact role of MEF 2A on the progression of atherosclerosis remains unclear. Therefore, we hypothesized that inhibition of its activity might affect the progression of atherosclerotic plaques in mice models. Homozygous MEF 2a–/–mice die suddenly within one week after birth, thus we used shRNA knockdown approach[3].

Atherosclerosis is a chronic inflammatory disease of the arterial wall, characterized by accumulation of lipids and macrophage-derived foam cells in the subendothelial space [20,21]. It is known that the inflammatory process contributes significantly to the initiation, progression and rupture of atherosclerotic plaques[20,22]. RNAi is a clinically feasible method to down-regulate the expression of target genes efficiently and selectively[15]. In our current work, silencing MEF 2A dramatically reduced mRNA and protein expression of MEF 2A in atherosclerotic plaques. Carotid plaques of the MEF 2A shRNA group showed lower collagen content, higher cross sectional plaque area, increased MCP-1 MMP-8 and TNF-α gene expression, and weaker fibrous caps than the scramble control and NC groups, indicating enhanced plaque vulnerability. Major clinical complications occur when atherosclerotic lesions evolve into complex, unstable forms.

Knockdown of MEF 2A in plaque increased the plasma concentration of pro-inflammatory cytokines MCP-1, MMP-8 and TNF-α in mice, implicating that MEF 2A interacts with other pro-inflammatory cytokines[1,14]. MCP-1 is an essential chemokine responsible for the recruitment of monocytes to inflammatory lesions[14,23]. Macrophages are the most significant source of inflammatory cytokines in the atherosclerotic plaques, such as MMP-8, TNF-α and IL-6[14,23]. High levels of pro-inflammatory cytokines may therefore favor the development of vulnerable plaques. MMP-8 possesses proteolytic activity on matrix proteins particularly type I collagen and promotes weakening of the fibrous cap[24]. Reports have shown that MMP overexpression was positively associated with the destruction of the extracellular matrix at the shoulders of plaques[25]. Knockdown of MEF 2A enhanced MMP-8 expression and decreased collagen content in the carotid plaques, which was in line with previous studies indicating that atherosclerosis in the MMP-8 deficient mice had increased collagen content [24–26]. These pro-inflammatory cytokines are known to contribute to vascular inflammation and plaque destabilization[26]. Moreover, we observed remarkable decrease in the collagen content and fibrous cap thickness in the plaques of the MEF 2A shRNA group, suggesting increased plaque vulnerability. Collectively, our results indicated that knockdown of MEF 2A increased the expression of pro-inflammatory cytokines, thereby contributing to pro-atherogenic and pro-inflammatory in facilitating atherosclerosis [32–36].

MEF 2A is expressed at high levels in the endothelium of coronary arteries[27,28]. Silencing MEF 2A may lead to a defective or abnormal vascular endothelium, which may promote the recruitment of monocytes to the subendothelial space and expose the subendothelial matrix to the vulnerable blood. This would make the artery wall be more prone to inflammation and thrombosis, ultimately leading to the development of atherosclerosis or myocardial infarction[4,29–31].

In summary, lentivirus-mediated shRNA effectively knocked down MEF 2A gene expression in APOE KO mice, resulting in increased plaque area and decreased plaque stability, independent of the plasma lipoprotein profile. Our findings underlined the importance of MEF 2A in atherosclerosis, which making MEF 2A to be a biomarker or a possible therapeutic target in atherosclerosis.
